# A Huge Penile Fibroepithelial Polyp Treated with Partial Penectomy: A Case Report and Review of the Literature

**DOI:** 10.1155/2020/6976254

**Published:** 2020-09-08

**Authors:** Adel Alrabadi, Sohaib Alhamss, Yasmeen Z. Qwaider, Saddam Al Demour

**Affiliations:** Department of Special Surgery, Division of Urology, School of Medicine, The University of Jordan, Amman, Jordan

## Abstract

Fibroepithelial polyps are benign tumors of mesodermal origin that usually arise on the surface of the skin and to a lesser extent in the urinary tract; however, their presence on the penis is extremely unusual. We report the case of a 73-year-old male with an extremely large broad-based penile fibroepithelial polyp (FEP) involving the penile shaft and glans penis associated with chronic condom catheter use and that was treated with partial penectomy. A review of the literature is included to highlight the rarity of this case. To the best of our knowledge, this is the largest mass of its kind to be reported on the penis.

## 1. Introduction

Fibroepithelial polyps (FEPs) are benign neoplasms that are mesodermal in origin. Histologically, they have a characteristic squamous epithelial surface with an underlying fibrovascular stroma, and definitive histology is needed to rule out malignancy [1]. We hereby present a case of a huge FEP on the glans penis associated with long-term condom catheter use.

## 2. Case Report

A 73-year-old male patient presented to our clinic complaining of a painless penile mass which has appeared 4 years ago and gradually been increasing in size. The patient has been known to have urinary urge incontinence for the last 17 years following spinal surgery. Since then, the patient has been using a condom catheter.

As the growth of the mass accelerated over the last 4 months, it became harder for the patient to have good hygiene and the condom could not fit anymore for the huge mass, and so he started to design special plastic bags for that purpose. In addition, the mass itself was becoming a source of a very bad odor. The patient has been complaining of erectile dysfunction, stool incontinence, and bilateral lower limb weakness since the time of spine surgery. The patient has a good urinary stream and has neither dysuria nor hematuria. He reported neither the presence of similar skin lesions, previous penile surgeries (apart from circumcision during childhood), nor a history of recent travels. The patient denied using penile constrictive rings or vacuum devices. There was no history of trauma. He was not taking medication.

**Figure 1:**
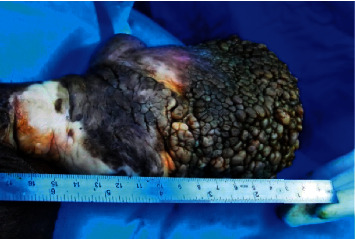
Giant fibroepithelial mass measuring at 13 cm on the ventral aspect of the shaft and glans penis.

**Figure 2:**
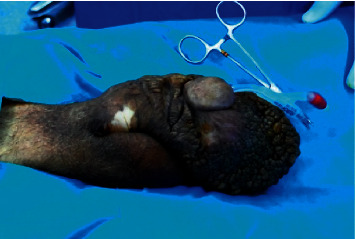
Giant fibroepithelial mass measuring at 13 cm on the ventral aspect of the shaft and glans penis.

On examination, the patient was having a huge (15 × 10 × 8 cm), nontender, “grape-like” penile firm mass originating from the ventral aspect of the penile shaft and glans penis (Figures 1 and 2). The lesion had a very broad base with no stalk. An extremely bad odor was originating from the mass. There was no ulcer and no discharge. The meatal opening, scrotal skin, and both testicles were all normal. The inguinal lymph nodes were not palpable. No other similar skin lesions were found.

Upon presentation, the serum creatinine level was 2.3 mg/dl. Urine analysis and culture showed the presence of urinary tract infection. The nonenhanced urinary tract computed tomography scan showed bilateral mild hydroureteronephrosis down to the urinary bladder. Ultrasonography showed a postvoid residual volume of 350 cc. 18-French Foley's catheter was inserted easily and kept in situ. The patient was admitted to the hospital and managed with intravenous fluids and antibiotics. His creatinine became 1.18 mg/dl.

Cystourethroscopy was performed and showed a normal-looking urethra, enlarged prostate, and severe trabeculations of the urinary bladder. At the same time, an incisional biopsy was performed which showed a benign fibroepithelial polyp with no evidence of malignancy. The situation was discussed with the patient and his family. Partial penectomy with excision of the whole mass was performed under general anesthesia. As the lesion was having a very broad base, it was not possible to excise the mass with preserving the penis. The postoperative course was uneventful. Follow-up for two years showed no recurrence.

**Figure 3:**
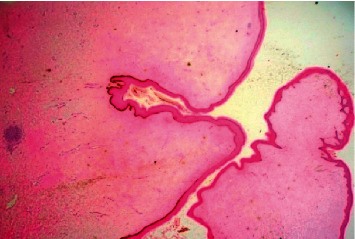
A hematoxylin and eosin stain of the fibroepithelial polyp showing a verrucous surface covered by acanthotic stratified squamous epithelium with hyperkeratosis. The stroma is hypocellular and collagenous.

**Figure 4:**
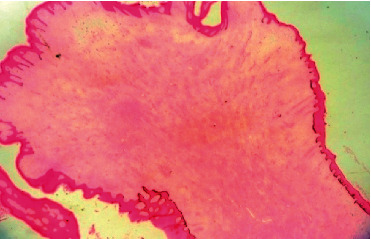
A hematoxylin and eosin stain of the fibroepithelial polyp showing a verrucous surface covered by acanthotic stratified squamous epithelium with hyperkeratosis. The stroma is hypocellular and collagenous.

**Figure 5:**
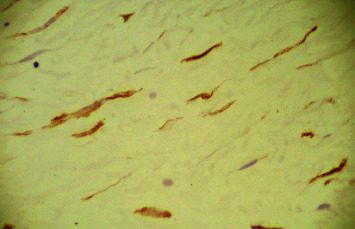
FEP stromal cells positive for CD34 staining (immunochemistry 200X).

The final histopathology of the specimen confirmed the previous diagnosis of a benign fibroepithelial polyp with no evidence of malignancy. It showed a hypocellular collagenized, edematous, and vascular stroma. The stroma contained patchy perivascular lymphocytic aggregates. Furthermore, few smooth muscle cell strands were noted. The surface was covered by benign acanthotic stratified squamous epithelium (Figures 3 and 4). Stromal cells were positive for CD34 staining (Figure 5).

## 3. Discussion

We have performed a review of the English-written literature using PubMed and Scopus looking for these terms: “fibroepithelial polyp” and “penile fibroepithelial polyp”. The references were reviewed from the available papers and studied. At the end, 25 papers were selected.

To the best of our knowledge, the literature describes only 25 cases of FEPs on the surface of the penis. The age reported in the studies ranged from 25 to 97 years, with the exception of 5 cases reported in children. Eleven of the adult cases, which constitute the majority, reported the long-term use of a condom catheter. 8 adult cases denied the use of the condom catheter. The remaining adult cases practiced male genital hanging kung fu [2]. Other possible causes for the rise of these polyps could be phimosis [1, 3] or the use of a cotton cloth for urinary incontinence [4].

It should also be noted that 17 of the cases occurred on the glans penis, making it the most common site. Other sites of manifestation were either on the frenulum, ventral surface of the penis, or penoscrotal junction (Table 1).

The overall prognosis of penile FEPs is good, with only 1 of the 25 cases transforming into squamous cell carcinoma [1]. Also, recurrence of the mass was rare with only 2 cases reappearing in less than 3 years (Table 1).

FEPs are benign tumors that arise from the mesoderm [18]. They mostly occur on the surface of the skin, specifically the axillae, neck, and eyelids [7]. In the urinary tract, they are mostly seen in the ureter, renal pelvis, and rarely in the posterior urethra or bladder [17] while their appearance on the penis is a rarity.

The etiology of penile FEPs is generally unknown but has been strongly linked to chronic, improper use of condom catheters [12]. It was hypothesized that pressure from condom catheters results in a reactive process and a decrease in vascular and lymphatic drainage [2, 4]. This theory may also explain the development of the FEP in Tsai et al.'s case report on a patient who practiced pressure-producing male genital hanging kung fu [2]. However, it does not explain the development of similar masses in children and in adults who used noncondom catheter.

Potentially, these FEPs could arise from the irritation of the glans [4], not only by direct pressure but by contact irritation through poor hygiene or cotton cloths.

In relation to the histopathology, all pathology reports from the previously reported cases similarly stated the characteristic fibrovascular edematous stroma covered by keratinized squamous epithelium [1–17]. Also, several authors stated the presence of mast cells [3, 13] and lymphocytes [3, 10, 12]. Our pathological findings were in conjunction with the findings of the literature.

In this reported case, the chronic use of a condom catheter by the patient might have predisposed to the development of the giant mass. It is important to note that the poor quality of hygiene the patient maintained might have played a significant role as well.

It is worth mentioning that upon the revision of the related literature, the current case we present has the largest mass recorded and at a maximum diameter of 15 cm. Partial penectomy was indicated in the case of our patient due to the replacement of the penile shaft and surrounding skin with the mass. Local complete excision of the mass with preserving the penis was impossible for us. We believe this procedure has never been performed in the case of a penile FEP according to the published data.

Finally, the postoperative course of the patient was uneventful; hence, we conclude that wide local excision and partial penectomy can be a good and satisfactory treatment option in the case of large polyps such as this. Additionally, our patient might solidify the theory that chronic, improper use of a condom catheter may predispose to the development of penile FEP. The role of hygiene should be noted as well and mentioned to all patients using condom catheters.

## 4. Review of the Literature

### 4.1. Methods

We underwent a review of the English-written literature using PubMed looking for the term: “penile fibroepithelial polyp”. Besides, we reviewed the references of the available articles.

### 4.2. Results

About 25 cases of a penile fibroepithelial polyp were reported in the literature (Table 1).

## Figures and Tables

**Table 1:** Review of the literature, cases of penile fibroepithelial polyps.

Authors	Age	Site	Size	Duration	Condom catheter use	Treatment	Follow-up	Recurrence	Malignancy
Yildirim et al. [5]	4	Glans	7 × 6 mm	NA	No	Local excision + circumcision	NA	NA	No
Fetsch et al. [6]	25	Glans	2.5 cm	Years	Yes	Local excision	13 years 8 months	Local recurrence <1 year. No evidence of disease at 12 years 8 months	No
Fetsch et al. [6]	29	Glans	3.4 cm	6 months	Yes	Local excision	24 years	No recurrence	No
Fetsch et al. [6]	32	Glans	2.5 cm	6 months	Yes	Local excision	1 month	No data	No
Fetsch et al. [6]	40	Glans	2 cm	10 years	Yes	Local excision	8 years 10 months	No recurrence	No
Fetsch et al. [6]	45	NA	3.5 cm	NA	NA	Local excision	Lost to follow-up	NA	No
Fetsch et al. [6]	52	Glans	7.5	1 year	No, paraphimosis	Local excision	6 years 2 months	No	No
Fetsch et al. [6]	58	Glans and prepuce	2.5 cm	NA	Yes	Local excision	3 years 10 months	Local recurrence at 3 years 7 months. No evidence of disease at 3 months (recurrence 2 lesions: 0.9 cm and 3 cm)	No
Emir et al. [7]	97	Distal ventral skin of the penis	5 × 3 cm	Less than 2 years	No	Local excision	NA	NA	NA
Al Awadhi et al. [8]	43	Ventral aspect of penis	4 cm	1 year	Yes (14 years)	Local excisional biopsy	NA	NA	No
Tsai et al. [2]	50	Glans	6.5 × 5 cm	5 years	No (male genitalia hanging kung fu)	Local excision	NA	NA	No
Turgut et al. [9]	59	Ventral aspect of penis	6 × 4.5 cm	10 years	Yes	Wide local excision and anticholinergic agent therapy was begun	1 year	No recurrence	No
Kampanatais et al. [1]	78	Glans	4.5	NA	No (phimosis)	Local excision + circumcision	6 months	No	Yes (SCC)
Pena et al. [10]	63	Glans	3 × 2.5 × 2 cm	NA	No	Local excision	6 months	No	No
Hyun et al. [11]	18 months	Penoscrotal junction	2.9 cm	18 months	No	Local excision	NA	NA	No
Banerji et al. [12]	42	Ventral aspect of the penis	8 × 5 cm	10 years	Yes	Local excision	NA	NA	No
Kim et al. [13]	45	Glans	6 × 3 × 3 cm	NA	Yes	Local excision	12 months	No	No
Mason et al. [3]	36	Glans (frenulum)	1.1 × 1.4 × 2.6 cm	1 year	Yes	Local excision	7 months	No	No
Kampanatais et al. [1]	35	Glans	7 cm	15 months	No	Local excision	60 months	No	No
Rodriguez Collar et al [14]	39	Frenulum	NA	5 months	No	Local excision + circumcision	12 months	No	No
Yan et al. [15]	62	Glans extending to frenulum	7 × 5 × 3 cm	11 years	No	Local excision + circumcision	NA	No	No
Goyal et al. [4]	38	Glans	3.5 × 3 × 2 cm	6 months	No, cotton cloth	Local excision	6 months	No	No
Sencan et al. [16]	6 months	Glans	6 × 7 mm	4 months	No	Local excision	1 year	No	No
Prashant et al. [17]	3	Glans	5 mm × 6 mm	NA	No	Local excision	1 year	No	No
Prashant et al. [17]	4	Glans	NA	NA	No	Local excision	NA	No	No

## Data Availability

The data used to support the study are included within the article.
